# Phosphoglycerate kinase 1 silencing by a novel microRNA microRNA-4523 protects human osteoblasts from dexamethasone through activation of Nrf2 signaling cascade

**DOI:** 10.1038/s41419-021-04250-1

**Published:** 2021-10-19

**Authors:** Jin-qian Liang, Zhen-tao Zhou, Lin Bo, Hai-ning Tan, Jian-hua Hu, Ming-sheng Tan

**Affiliations:** 1 Department of Orthopaedics, Peking Union Medical College Hospital, Beijing, China; 2 Department of Orthopedics, The Second Affiliated Hospital of Soochow University, Suzhou, China; 3 Department of Rheumatology, The Second Affiliated Hospital of Soochow University, Suzhou, China; 4 Spinal Surgery, Sino-Japanese Friendship Hospital, Beijing, China

**Keywords:** RNA, Stress signalling

## Abstract

Nuclear-factor-E2-related factor 2 (Nrf2) cascade activation can ameliorate dexamethasone (DEX)-induced oxidative injury and death in human osteoblasts. Phosphoglycerate kinase 1 (PGK1) depletion is shown to efficiently activate Nrf2 signaling by inducing methylglyoxal modification of Kelch-like ECH-associated protein 1 (Keap1). We here identified a novel PGK1-targeting microRNA: microRNA-4523 (miR-4523). RNA fluorescent in situ hybridization, RNA pull-down, and Argonaute-2 RNA immunoprecipitation results confirmed a direct binding between miR-4523 and *PGK1* mRNA in primary human osteoblasts and hFOB1.19 osteoblastic cells. Forced overexpression of miR-4523, using a lentiviral construct, robustly decreased *PGK1* 3′-UTR (untranslated region) luciferase activity and downregulated its expression in human osteoblasts and hFOB1.19 cells. Furthermore, miR-4523 overexpression activated the Nrf2 signaling cascade, causing Keap1–Nrf2 disassociation, Nrf2 protein stabilization, and its nuclear translocation as well as transcription activation of Nrf2-dependent genes (*NQO1*, *GCLC,* and *HO1*) in human osteoblasts. By expressing a UTR-null PGK1 construct, miR-4523 overexpression-induced Nrf2 cascade activation was however largely inhibited. Importantly, DEX-induced reactive oxygen species production, oxidative injury, and cell apoptosis were significantly attenuated by miR-4523 overexpression in human osteoblasts and hFOB1.19 cells. Such actions by miR-4523 were abolished by Nrf2 shRNA or knockout, but mimicked by PGK1 knockout (using CRISPR/Cas9 method). In PGK1 knockout human osteoblasts, miR-4523 overexpression failed to further increase Nrf2 cascade activation and offer osteoblast cytoprotection against DEX. Significantly, miR-4523 is downregulated in human necrotic femoral head tissues of DEX-taking patients. Together, PGK1 silencing by miR-4523 protected human osteoblasts from DEX through activation of the Nrf2 signaling cascade.

## Introduction

For patients with chronic inflammatory and auto-immune diseases, dexamethasone (DEX) and other glucocorticoids are commonly prescribed [1]. However, prolonged/overdose usage of DEX could lead to significant cytotoxicity to human osteoblasts, serving as a primary cause of osteoporosis (or even osteonecrosis) [2, 3]. In vitro studies have shown that DEX can induce direct and profound oxidative injury and cytotoxicity to cultured human osteoblasts and osteoblastic cells [2, 4–7]. Our group is dedicated to understanding the pathological mechanisms of DEX-induced osteoblast injury and exploring potential targeted intervention strategies [8, 9].

Activation of nuclear-factor-E2-related factor 2 (Nrf2) cascade is essential to inhibit oxidative stress and cell injuries [10, 11]. Studies have shown that Nrf2 activation, using genetic strategies or pharmacological methods, can efficiently protect osteoblasts/osteoblastic cells from DEX-induced oxidative injury [6, 7, 9, 12–15]. Under resting state, Nrf2 protein mainly stays in the cytosol and directly binds to its suppressor protein Kelch-like ECH-associated protein 1 (Keap1) [16–18]. Through the E3 ubiquitin ligase Cullin 3, Keap1 will dictate Nrf2 protein degradation. Different stimuli will cause various post-translational modifications of Nrf2 and/or Keap1, leading to Keap1–Nrf2 disassociation. This will free Nrf2 from ubiquitin-mediated degradation and cause Nrf2 protein stabilization [16–18]. Stabilized Nrf2 protein will then translocate to cell nuclei and bind to antioxidant response element (ARE) and small MAF proteins, causing transcription activation and expression of multiple Nrf2-dependent genes [16–18]. The majority of Nrf2 cascade genes are antioxidant genes and detoxifying enzymes, including heme oxygenase-1 (*HO1*), NAD(P)H quinone oxidoreductase 1 (*NQO1*), γ-glutamyl cysteine ligase catalytic subunit (*GCLC*), and the modified subunit *GCLM* [16–18].

Previous studies have discovered that phosphoglycerate kinase 1 (PGK1) is a key negative regulator of Nrf2 [19]. PGK1 silencing or blockage will cause accumulation of reactive metabolite methylglyoxal. The latter could form a characteristic methylimidazole crosslink between proximal cysteine and arginine residues (MICA) in Keap1 [19, 20], leading to Keap1 dimerization, Keap1–Nrf2 separation, and Nrf2 activation [19, 20]. Our previous study has discovered that shRNA-induced silencing or CRISPR/Cas9-induced knockout of PGK1 robustly activated Nrf2 signaling cascade and potently inhibited DEX-induced oxidative injury in human osteoblasts [9]. These results implied that PGK1 silencing could offer significant osteoblast cytoprotection against DEX via activation of the Nrf2 cascade.

MicroRNAs (miRNAs) are conserved small non-coding RNAs (ncRNAs) with ~21–23 nucleotides long [21–23]. miRNAs regulate gene expression through direct binding to the 3′-untranslated region (3′-UTR) of targeted mRNAs, leading to mRNA translation inhibition and/or degradation [21–23]. Recent studies have shown that various miRNAs could activate the Nrf2 signaling cascade to inhibit DEX-induced oxidative injury in osteoblasts and osteoblastic cells [6, 7, 24]. We hypothesized that targeting PGK1 by specific miRNAs should activate Nrf2 signaling and offer osteoblast cytoprotection against DEX. We here identified microRNA-4523 (miR-4523) as a novel and specific PGK1-targeting miRNA. Our results showed that miR-4523 silenced PGK1 and protected human osteoblasts from DEX by activation of the Nrf2 cascade.

## Materials and methods

### Chemicals, reagents, and antibodies

DEX, polybrene, puromycin, JC-1, and MTT were obtained from Sigma-Aldrich Chemicals (St. Louis, MO). Antibodies for HO1 (#70081), NQO1 (#3187), Nrf2 (#12721), Keap1 (#8047), α-Tubulin (#2125), and Lamin B1 (#13435) as well as cleaved-poly (ADP-ribose) polymerase (PARP, #5625) and cleaved-caspase-3 (#9664) were provided by Cell Signaling Tech (Shanghai, China). The anti-GCLC antibody (ab55435) was purchased from Abcam (Shanghai, China). The anti-PGK1 (sc-130335) antibody was from Santa Cruz Biotech (Santa Cruz, CA). Cell culturing reagents, including fetal bovine serum (FBS), Dulbecco’s Modified Eagle Medium (DMEM), Roswell Park Memorial Institute, and antibiotics, were purchased from Gibco-BRL (Nanjing, China). RNA reagents, Annexin V, CellROX, propidium iodide (PI), and cell transfection reagents (Lipofectamine 3000 and others) were all provided by Thermo-Fisher Invitrogen (Shanghai, China). All mRNA primers, viral constructs, and sequences were synthesized by Shanghai Genechem Co. (Shanghai, China) unless otherwise mentioned.

### Cell culture

hFOB1.19 human osteoblastic cells were from Dr. Cui at Nantong University and were cultured in DMEM medium plus 8% FBS [4]. Isolation and primary culturing of human osteoblasts were described previously [2, 25]. Short tandem repeat profiling, population doubling time, and morphology were monitored regularly to verify cells. Primary human osteoblasts were cultured for <12 passages. The protocols were according to the principles of the Declaration of Helsinki, with approval from the Ethics Committee of Peking Union Medical College Hospital.

### Quantitative real-time PCR (qRT-PCR)

TRIzol reagent (Invitrogen Thermo-Fisher, Shanghai, China) was utilized to extract total RNA. cDNA was synthesized via reverse transcription by a Superscript II reverse transcriptase kit (Invitrogen). A SYBR GREEN PCR Master Mix kit was utilized for qRT-PCR assays under the ABI Prism 7900 Fast Real-Time PCR system. For data quantification, the melting temperature was calculated. *GAPDH* was tested the reference gene/internal control. Data quantification was through the 2^−∆∆*C*t^ method. Expression of mature miR-4523 was normalized to *U6* RNA. Primers were listed below: *U6* mRNA forward: 5′-CTCGCTTCGGCAGCACATATACT-3′ and reverse: 5′-ACGCTTCACGAATTTGCGTGTC-3′. miR-4523 forward: 5′-GACCGAGAGGGCCTCGGC-3′ and reverse: 5′- GAACATGTCTGCGTATCTC-3′. Other primers were listed in our previous study [9].

### Western blotting and co-immunoprecipitation (Co-IP)

The detailed procedures of Western blotting and Co-IP assays were described elsewhere [26, 27]. Data quantification was through an ImageJ software (NIH, USA), the total gray of each protein band was normalized to that of the loading control.

### Exogenous overexpression or silencing of miR-4523

For overexpression, the full-length miR-4523 precursor (pre-miR-4523) sequence was synthesized by Genechem and was inserted into a GV369 lentiviral vector (Genechem). The construct, along with the lentivirus package Helper plasmids, were co-transfected to HEK-293T cells, generating pre-miR-4523-expressing lentivirus (“lv-pre-miR-4523”). The viruses were then filtered, enriched (MOI = 10), and transduced to osteoblasts (cultured in polybrene-containing complete medium). Afterwards, osteoblasts were returned back to complete medium. Puromycin (2.5 μg/mL) was added to select stably expressing cells. For miR-4523 silencing, the sequence that encodes the pre-miR-4523 antisense (antagomR-4523) was sub-cloned into the GV369 lentiviral vector to generate lv-antgomiR-4523 construct (Genechem). The construct was stably transduced to human osteoblasts similarly. The lentiviral microRNA control (“lv-miRC”) and microRNA antisense control (“lv-antaC”) were utilized as controls.

### Transfection of miR mimic

Human osteoblasts or hFOB1.19 cells were initially seeded into six-well plates (at 60% confluence) and transfected with the applied miR mimic (500 nM for 24 h, two rounds) using Lipofectamine 3000 [28].

### PGK1 3′-UTR activity

Using the previously-described protocols [29], a firefly-luciferase reporter vector pGL4.13 (luc2/SV40) with the *PGK1* 3′-UTR (containing miR-4523′s putative binding sites at position 898–905) was synthesized by Shanghai Genechem. The primary human osteoblasts or hFOB1.19 osteoblastic cells were transfected with the plasmid, along with the Renillaluciferase reporter vector and pRL-SV40 (Promega, Shanghai, China) [29] by Lipofectamine 3000 (Invitrogen). Cells were then subjected to applied genetic modifications, and the *PGK1* 3′-UTR luciferase reporter activity was examined through a Promega kit [28].

### ARE reporter assay

Primary human osteoblasts or hFOB1.19 osteoblastic cells were seeded into a six-well plate (at 0.8 × 10^5^ cells/well), and transfected with an ARE-inducible firefly-luciferase vector (provided by Dr. Jiang at Nanjing Medical University [30]). Cells were then subjected to applied genetic modifications and cell lysates were subjected to tests of the luciferase activity under a luminescence machine.

### UTR-null PGK1 expression

The GV369 lentiviral construct encoding the 3′-UTR-null *PGK1* was generated by Genechem (Shanghai, China). The construct was transduced to primary human osteoblasts. Stable cells were selected by puromycin. PGK1 expression was verified by Western blotting assays.

### RNA pull-down

RNA pull-down assay was performed by a Pierce Magnetic RNA Pull-Down Kit using the described protocols [31, 32]. In brief, human osteoblasts or hFOB1.19 osteoblastic cells were transfected with biotinylated-miR-4523 mimic or control mimic (Genechem, 500 nmol/L) for 36 h, and the cells were harvested [32]. Total cell lysates were incubated with streptavidin-coated magnetic beads to pull-down the biotin-captured RNA complex [31]. *PGK1 mRNA* was then examined through qRT-PCR assays.

### RNA fluorescent in situ hybridization (FISH)

RNA-FISH was carried out through fluorescent in situ hybridization kit (RiboBio, Guangzhou, China). In brief, FITC-labeled miR-4523 probes (in green fluorescence. RiboBio) and Cy3-labeled *PGK1* mRNA probes (in red fluorescence, RiboBio) were transduced to primary human osteoblasts or hFOB1.19 osteoblastic cells (at 37 °C for 12 h). Cells were then rinsed and observed under a fluorescence microscope (Leica, Shanghai, China).

### RNA immunoprecipitation (RNA-IP)

Primary human osteoblasts or hFOB1.19 cells were cultured in 10-cm diameter dishes and were lysed in RIP lysis buffer (Beyotime Biotechnology, Wuxi, China). Total cell extracts, 800 μg lysates in 800 μL lysis buffer per treatment, were pre-cleared and then incubated with magnetic beads conjugated with the anti-Argonaute-2 antibody (Ago2, Santa Cruz Biotech) overnight. Next, the beads were washed and incubated with Proteinase K. The purified RNA was subjected to qRT-PCR analysis and normalized to the “Input” controls.

### Nrf2 silencing

The Nrf2 shRNA lentiviral particles (Santa Cruz Biotech) were added to cultured human osteoblasts (cultured in polybrene-contain complete medium). After selection by puromycin stable osteoblasts were established. Nrf2 silencing was always verified by qRT-PCR and Western blotting assays.

### CRISPR/Cas9-induced gene knockout (KO)

The primary human osteoblasts were transduced with a CRISPR/Cas9 PX458-PGK1-KO-GFP construct (see our previous study [9]) or a CRISPR/Cas9-Nrf2-KO-GFP construct (see our previous study [9]). Fluorescence-activated cell sorting (FACS) was applied to sort GFP-positive osteoblasts. Osteoblasts were distributed to 96-well plates and subjected to PGK1/Nrf2-KO screening by qRT-PCR and western blotting assays. Control osteoblasts were transduced with the CRISPR/Cas9 PX458-GFP construct with scramble non-sense sgRNA (“Cas9-C”).

### Assaying of reactive oxygen species (ROS) contents

Primary human osteoblasts or HFOB1.19 osteoblastic cells, with applied genetic modifications, were seeded into six-well plates at 60% confluence. Osteoblasts were stained with CellROX (7.5 μg/mL) and washed with PBS. The representative CellROX fluorescence images were taken by Leica confocal microscope. The CellROX intensity was measured under a spectrofluorometer (F-7000, Hitachi, Japan) at 625 nm (red fluorescence).

### DNA damage

Primary human osteoblasts or hFOB1.19 osteoblastic cells with applied genetic modifications were seeded into 96-well plates (at 4000 osteoblasts per well). A single-strand DNA (ssDNA) ELISA kit (EMD Millipore, Shanghai, China) was utilized to examine ssDNA levels, with ELISA absorbance (OD) examined at 405 nm.

### Other cell functional assays

MTT assay of cell viability, JC-1 assaying of mitochondrial depolarization, thiobarbituric acid reactant (TBAR) assay of lipid peroxidation, apoptosis-related assays, including Annexin V-PI FACS, nuclear TUNEL staining, Histone DNA ELISA, and caspase-3/-9 activity assays, were described in detail in our previous studies [8, 9] and also in elsewhere [33, 34].

### Human tissue collection and analyses

As described in our previous study [9], human necrotic femoral head tissues and surrounding normal femoral head tissues were from 30 written-informed consent DEX-treated patients (24 female and 6 male) undergoing femoral head resection. Seven patients received short-term high usage of DEX and 23 patients received long-term DEX treatment. Patients were at femoral head necrosis stage II or necrosis early stage III (with slight collapse at the femoral head). Human tissues were obtained freshly at the time of surgery and stored in the liquid nitrogen before further analyses. Expressions of miR-4523 and listed mRNAs in tissue lysates were tested by qRT-PCR assays. Protocols were according to the principles of the Declaration of Helsinki, with approval from the Ethics Committee of Peking Union Medical College Hospital.

### Statistical analyses

All quantitative data were with normal distribution and presented as means ± standard deviation (SD). Statistical analyses were performed through the one-way analysis of variance together with the multiple comparisons with Bonferroni’s post hoc test (SPSS 23.0; SPSS Co., Chicago, IL). When comparing two treatment groups a two-tailed unpaired *T* test (Excel 2007) was utilized. *P* values <0.05 were considered statistically significant.

## Results

### miR-4523 silences PGK1 and activates Nrf2 signaling cascade in human osteoblasts

**Fig. 1:**
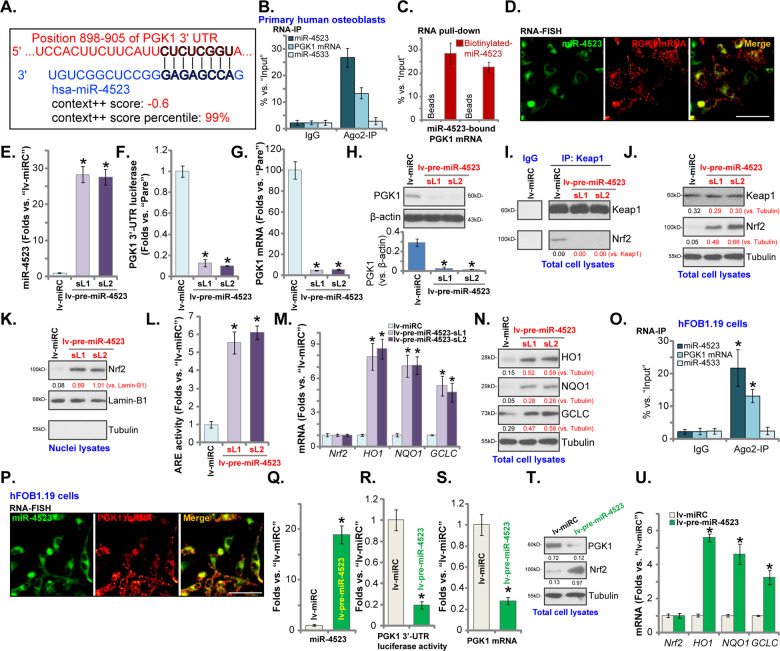
miR-4523 silences PGK1 and activates Nrf2 signaling cascade in human osteoblasts. miR-4523 putatively targets *PGK1* mRNA 3′-UTR (at position 898–905) (**A**). RNA immunoprecipitation (RNA-IP) demonstrated that the Ago2 protein immunoprecipitated with endogenous *PGK1* mRNA and miR-4523 (but not miR-4533, a negative control) in human osteoblasts (**B**). RNA pull-down assay results confirmed a direct binding between biotinylated-miR-4523 and *PGK1* mRNA in primary human osteoblasts (**C**). RNA fluorescence in situ hybridization (FISH) confirmed miR-4523 (in green) and *PGK1* mRNA (in red) colocalization in primary human osteoblasts (**D**). Primary human osteoblasts were transduced with a lentiviral construct encoding the pre-miR-4523 (“lv-pre-miR-4523”) or control miR construct (lv-miRC), and stable cells established after puromycin selection; Expression of mature miR-4523 (**E**), listed mRNAs (**G** and **M**) and proteins (in total cell lysates and nuclear lysates, **H**, **J**, **K**, **N**) were tested by qRT-PCR and western blotting assays. The relative *PGK1* 3′-UTR luciferase reporter activity (**F**) and the relative ARE activity (**L**) were tested as well; Keap1–Nrf2 association was tested by co-immunoprecipitation (Co-IP) assays (**I**). Ago2 RNA-IP (**O**) and RNA-FISH (**P**) confirmed the direct binding between miR-4523 and *PGK1* mRNA in hFOB1.19 osteoblastic cells. Stable hFOB1.19 cells expressing lv-pre-miR-4523 or lv-miRC were established; Expression of mature miR-4523 (**Q**), listed mRNAs (**S** and **U**) and proteins (in total cell lysates, **T**) were tested by qRT-PCR and western blotting assays; The relative *PGK1* 3′-UTR luciferase reporter activity was tested as well (**R**). Data were presented as mean ± standard deviation (SD, *n* = 5). *P < 0.05 versus “lv-miRC” group. The experiments were repeated five times with similar results obtained. Scale bar = 100 μm (**D** and **P**).

TargetScan (V7.2, http://www.targetscan.org) was first consulted to predict miRNAs that can possibly bind to 3′-UTR of *PGK1*. Retrieved miRNAs were further verified using other miRNA databases, including miRBase, miRNAmap, and miRTarbase. Several candidate PGK1-targeting miRNAs with the context score percentage over 99% and the context^++^ score <−0.5 were retrieved [35], including microRNA-4523 (miR-4523), miR-6789-5p, miR-4999-3p, miR-6862-3p, miR-6784-3p. Each miRNA mimic was transfected to primary human osteoblasts, and their efficiency in silencing *PGK1* was analyzed. As shown in Figure S1, only transfection of the miR-4523 mimics resulted in potent *PGK1* mRNA downregulation. miR-4523 targets the 3′-UTR (position 898–905) of *PGK1* (Fig. 1A). The miR-4523-*PGK1* 3′-UTR binding context score percentage is 99% and the context^++^ score is −0.6 (TargetScan V7.2 [35], Fig. 1A). These parameters indicated a high probability of direct binding between the two [35].

To test whether miR-4523 and *PGK1* mRNA could bind to each other, we first employed a RIP assay. Results found that endogenous *PGK1* mRNA and miR-4523 were immunoprecipitated with an anti-Ago2 antibody in the primary human osteoblasts (Fig. 1B). The non-specific anti-IgG antibody failed to pull-down *PGK1* mRNA and miR-4523 (Fig. 1B). miR-4533, a negative control, also failed to immunoprecipitate with the Ago2 protein (Fig. 1B). In addition, RNA pull-down assay results demonstrated that the biotinylated-miR-4523 is directly associated with *PGK1* mRNA in primary human osteoblasts (Fig. 1C). Furthermore, FISH assay results demonstrated that miR-4523 (green fluorescence) and *PGK1* mRNA (green fluorescence) mainly colocalized in the cytosol of primary human osteoblasts (Fig. 1D). These results implied that miR-4523 and *PGK1* mRNA are directly associated with each other in human osteoblasts.

To test whether miR-4523 could affect *PGK1* mRNA expression, a lentiviral construct encoding the miR-4523 precursor sequence, lv-pre-miR-4523, was transduced to primary human osteoblasts. Two stable cell lines, lv-pre-miR-4523-sL1 and lv-pre-miR-4523-sL2, were established after puromycin selection. qRT-PCR assay results demonstrated that miR-4523 levels increased over 25 folds in lv-pre-miR-4523-expressing human osteoblasts (Fig. 1E). Conversely, the *PGK1* 3′-UTR luciferase activity was dramatically reduced (Fig. 1F). Importantly, the expression of *PGK1* mRNA (Fig. 1G) and protein (Fig. 1H) was significantly decreased as well.

Our previous study has shown that PGK1 depletion could induce Nrf2 cascade activation in human osteoblasts [9]. We thus tested Nrf2 signaling in miR-4523-overexpressed osteoblasts. Co-IP assay results showed that the Keap1–Nrf2 association was disrupted in lv-pre-miR-4523-expressing human osteoblasts (Fig. 1I), where Nrf2 protein levels were significantly elevated (Fig. 1J). Keap1 protein expression was however unchanged (Fig. 1J). When testing nuclear fraction lysate proteins, we found that Nrf2 protein translated to the nuclei of miR-4523-overexpressed osteoblasts (Fig. 1K). Moreover, the relatively ARE activity was significantly increased (Fig. 1L). Importantly, mRNA (Fig. 1M) and protein (Fig. 1N) expression of Nrf2-ARE-dependent genes, including *HO1*, *NQO1,* and *GCLC*, were significantly increased in osteoblasts with miR-4523 overexpression (Fig. 1M and N). *Nrf2* mRNA levels were however unchanged (Fig. 1M). These results showed that miR-4523 overexpression activated the Nrf2 cascade in human osteoblasts, causing Keap1–Nrf2 disassociation, Nrf2 protein stabilization, and its nuclear translocation, as well as transcription activation and expression of Nrf2-ARE-dependent genes.

We also tested whether miR-4523 exerted similar functions in hFOB1.19 osteoblastic cells. Ago2 RNA-IP results showed that that the Ago2 protein immunoprecipitated with miR-4523 and *PGK1* mRNA in hFOB1.19 cells (Fig. 1O). RNA-FISH results confirmed a direct binding between miR-4523 and *PGK1* mainly in the cytosol of hFOB1.19 cells (Fig. 1P). Similarly, lv-pre-miR-4523 was stably transduced to hFOB1.19 cells, resulting in robust miR-4523 overexpression (Fig. 1Q). *PGK1* 3′-UTR luciferase activity (Fig. 1R) and its mRNA (Fig. 1S) levels were dramatically decreased in lv-pre-miR-4523-expressed hFOB1.19 cells. Nrf2 protein stabilization (Fig. 1T) as well as increased mRNA expression of ARE-dependent genes (*HO1*, *NQO1,* and *GCLC*) (Fig. 1U) were detected as well in miR-4523-overexpressed hFOB1.19 cells. These results showed that miR-4523 silenced PGK1 and activated the Nrf2 signaling cascade in human osteoblasts.

### Nrf2 cascade activation by miR-4523 is due to PGK1 silencing in human osteoblasts

**Fig. 2:**
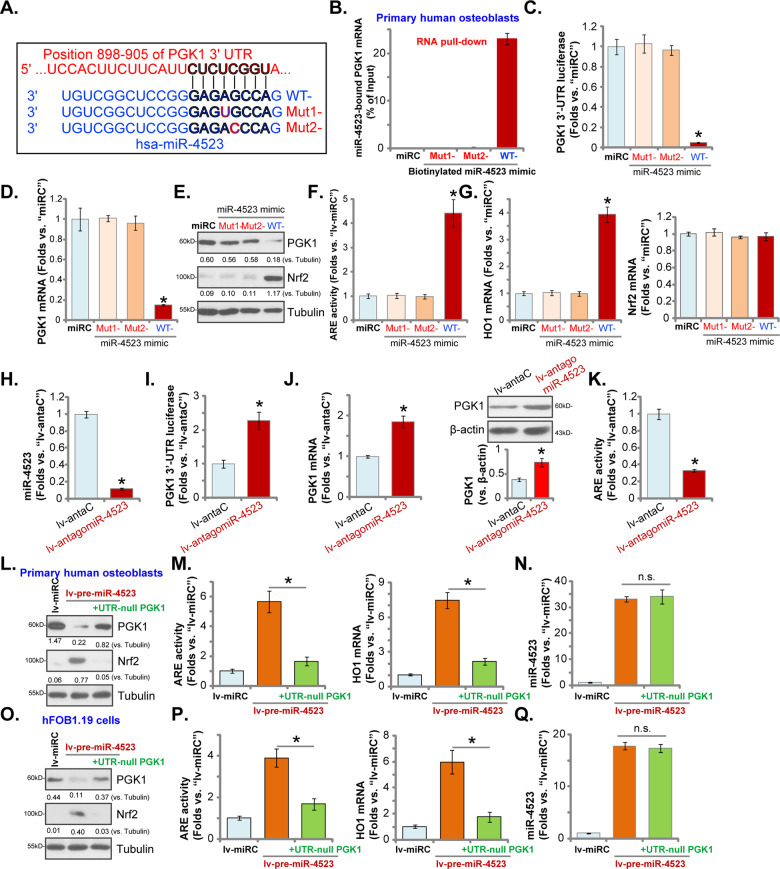
Nrf2 cascade activation by miR-4523 is due to PGK1 silencing in human osteoblasts. The sequences of the wild-type (WT) and mutant miR-4523 mimics were listed (**A**). RNA pull-down assay results confirmed the direct binding between biotinylated-WT-miR-4523 (but not the mutants) and *PGK1* mRNA in primary human osteoblasts (**B**). The primary human osteoblasts were transfected with applied WT or mutant miR-4523 mimics (500 nM for 24 h, two rounds), relative *PGK1* 3′-UTR luciferase activity (**C**), expressions of listed mRNAs (**D** and **G**) and proteins (**E**) as well as the relative ARE activity (**F**) were tested. Stable human osteoblasts expressing the lentiviral construct encoding the antisense sequence of miR-4523 (lv-antagomiR-4523) or the control construct (lv-antaC) were established and cultured. Expression of miR-4523 was tested (**H**). The *PGK1* 3′-UTR luciferase reporter activity (**I**) as well as its mRNA and protein expression (**J**) were tested, with the relative ARE activity (**K**) examined as well. Primary human osteoblasts (**L**–**N**) or hFOB1.19 osteoblastic cells (**O**–**Q**), expressing the lentiviral construct encoding the pre-miR-4523 (“lv-pre-miR-4523”), were further transduced with or without 3′-UTR-depleted PGK1 construct (“+UTR-null PGK1”). Control cells were transduced with the control miR construct (lv-miRC). Expression of listed proteins was tested by western blotting assays (**L** and **O**). The ARE activities, *HO1* mRNA levels (**M** and **P**), and miR-4523 expression (**N** and **Q**) were also tested. Data were presented as mean ± standard deviation (SD, *n* = 5). “miRC” stands for non-sense control miRNA mimic (**B**–**G**) *P < 0.05 versus “miRC” cells (**B**–**G**). *P < 0.05 versus “lv-antaC” cells (**H**–**K**). *P < 0.05 (**M** and **P**) “n.s.” stands for non-statistical difference (**N** and **Q**). The experiments were repeated five times with similar results obtained.

To further support that miR-4523 directly targets *PGK1* mRNA in human osteoblasts, two mutant miR-4523 mimics, containing mutations at the binding sites to *PGK1* 3′-UTR (Fig. 2A), were synthesized. The wild-type (WT) and mutant miR-4523 mimics were individually transfected to primary human osteoblasts. RNA pull-down assay results confirmed that only the WT miR-4523 mimic (biotinylated), but not the mutants, could associate with *PGK1* mRNA (Fig. 2B). Transfection of the WT miR-4523 mimics significantly decreased the *PGK1* 3′-UTR luciferase reporter activity (Fig. 2C) as well as its mRNA (Fig. 2D) and protein (Fig. 2E) expression, whereas the mutants were ineffective (Fig. 2C–E). Furthermore, the WT miR-4523 mimic provoked Nrf2 signaling cascade activation by inducing Nrf2 protein stabilization (Fig. 2E), ARE activity increase (Fig. 2F), and *HO1* mRNA expression (Fig. 2G) in human osteoblasts. *Nrf2* mRNA levels were however unchanged (Fig. 2G). The mutants were again invalid (Fig. 2E–G).

Next, a lentiviral construct encoding the antisense of miR-4523, or lv-antagomiR-4523, was stably transduced to primary human osteoblasts, resulting in potent miR-4523 silencing (verses human osteoblasts with lv-antaC control, Fig. 2H). As shown, lv-antagomiR-4523 increased *PGK1* 3′-UTR luciferase reporter activity (Fig. 2I) as well as its mRNA and protein expression (Fig. 2J) in human osteoblasts. Conversely, the relative ARE activity was downregulated (Fig. 2K). Therefore, miR-4523 silencing increased PGK1 expression, further supporting that miR-4523 is a *PGK1*-targeting miRNA.

To further support our hypothesis, a lentiviral construct encoding the 3′-UTR-depleted PGK1 (“UTR-null PGK1”) was transduced to lv-pre-miR-4523-expressing human osteoblasts, and it restored PGK1 expression (Fig. 2L). Importantly, lv-pre-miR-4523-induced Nrf2 protein stabilization (Fig. 2L), ARE activity increase (Fig. 2M) and *HO1* mRNA expression (Fig. 2M) were largely inhibited by the UTR-null PGK1. As expected, UTR-null PGK1 failed to affect miR-4523 expression in human osteoblasts (Fig. 2N). Similar results were obtained in hFOB1.19 osteoblastic cells. The UTR-null PGK1 restored PGK1 expression (Fig. 2O) and blocked Nrf2 cascade activation (Fig. 2O and P) in lv-pre-miR-4523-expressing hFOB1.19 cells. miR-4523 expression was once again unchanged (Fig. 2Q). These results implied that restoring PGK1 expression inhibited miR-4523 overexpression-induced Nrf2 signaling activation, suggesting that PGK1 silencing should be the primary mechanism of miR-4523-induced Nrf2 cascade activation in human osteoblasts.

### miR-4523 overexpression attenuates DEX-induced oxidative injury in human osteoblasts

**Fig. 3:**
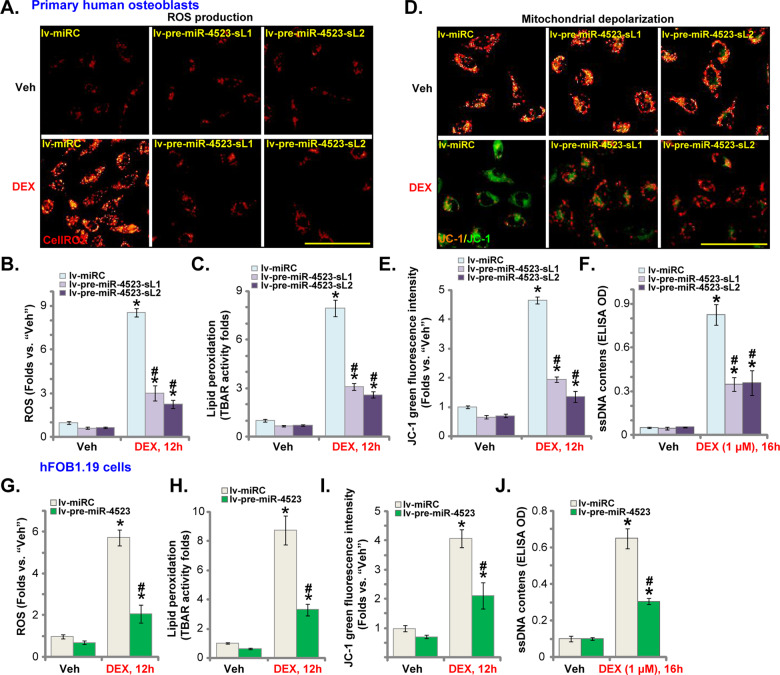
miR-4523 overexpression attenuates DEX-induced oxidative injury in human osteoblasts. Primary human osteoblasts (**A**–**F**) or hFOB1.19 osteoblastic cells (**G**–**J**), stably expressing a lentiviral construct encoding the pre-miR-4523 (“lv-pre-miR-4523”) or control miR construct (lv-miRC), were established. Cells were then treated with dexamethasone (DEX, 1 μM) or vehicle control (“Veh”) and cultured for applied time periods. Cellular ROS contents (**A**, **B**, and **G**), lipid peroxidation intensity (**C** and **H**), mitochondrial depolarization (**D**, **E**, and **I**), and single-strand DNA contents (ELISA OD, **F** and **J**) were tested by the assays mentioned in the text. Data were presented as mean ± standard deviation (SD, *n* = 5). *P < 0.05 versus “Veh” treatment in “lv-miRC” cells; ^#^***P*** < 0.05 versus “DEX” treatment in “lv-miRC” cells. The experiments were repeated five times with similar results obtained. Scale bar = 100 μm (**A** and **D**).

Studies have shown that DEX could induce ROS production and oxidative injury in osteoblasts [6, 9, 24, 36]. On the contrary, Nrf2 activation will significantly ameliorate DEX-induced oxidative injury and inhibited osteoblast cell death [6, 9, 24, 36]. We, therefore, analyzed whether miR-4523 could inhibit DEX-induced oxidative injury in human osteoblasts. Testing cellular ROS contents, by CellROX staining assays, demonstrated that DEX stimulation led to robust ROS production in lv-miRC-expressing control human osteoblasts (Fig. 3A, B). Increased lipid peroxidation (TBAR activity) was detected as well in DEX-treated lv-miRC control osteoblasts (Fig. 3C). Importantly, in lv-pre-miR-4523-expressing human osteoblasts, DEX-induced ROS production and lipid peroxidation were largely attenuated (Fig. 3A–C).

Furthermore, DEX induced significant mitochondrial depolarization (JC-1 green monomers accumulation, Fig. 3D, E) and DNA damage (ssDNA contents increase, Fig. 3F) in lv-miRC-expressing osteoblasts. Such actions by DEX were largely attenuated in miR-4523-overexpressed human osteoblasts (Fig. 3D–F). Similar results were obtained in hFOB1.19 osteoblastic cells, where lv-pre-miR-4523 largely ameliorated DEX-induced ROS production (CellROX intensity increase, Fig. 3G), lipid peroxidation (TBAR activity increase, Fig. 3H), mitochondrial depolarization (JC-1 green monomers accumulation, Fig. 3I), and ssDNA accumulation (Fig. 3J). These results showed that miR-4523 overexpression attenuated DEX-induced oxidative injury in human osteoblasts.

### miR-4523 overexpression ameliorates DEX-induced apoptosis in human osteoblasts

**Fig. 4:**
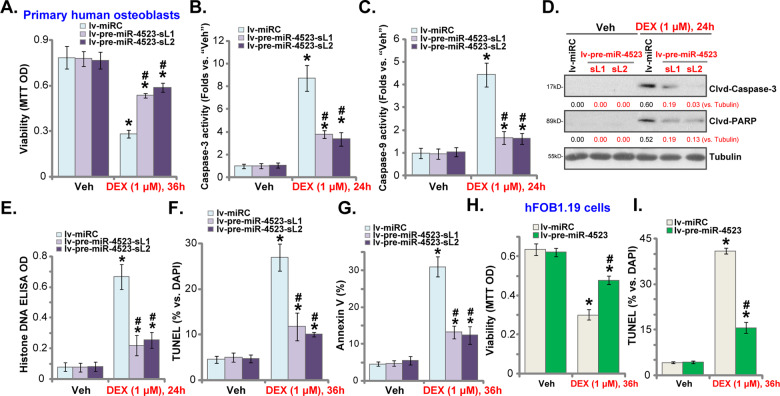
miR-4523 overexpression ameliorates DEX-induced apoptosis in human osteoblasts. Primary human osteoblasts (**A**–**F**) or hFOB1.19 osteoblastic cells (**G** and **H**), stably expressing a lentiviral construct encoding the pre-miR-4523 (“lv-pre-miR-4523”) or control miR construct (lv-miRC), were established. Cells were then treated with dexamethasone (DEX, 1 μM) or vehicle control (“Veh”), and cultured for applied time periods. Cell viability was tested by MTT assays (**A** and **H**). Caspase-3 and caspase-9 activities (**B** and **C**) as well as expression of apoptosis-associated proteins (**D**) and histone-bound DNA contents (**E**) were tested. Cell apoptosis was examined by nuclear TUNEL staining (**F** and **I**) and Annexin V FACS (**G**) assays, with results quantified. Data were presented as mean ± standard deviation (SD, *n* = 5). *P < 0.05 versus “Veh” treatment in “lv-miRC” cells; ^#^ ***P*** < 0.05 versus “DEX” treatment in “lv-miRC” cells. The experiments were repeated five times with similar results obtained.

Since miR-4523 overexpression largely attenuated DEX-induced ROS production and oxidative injury in human osteoblasts, we tested its effect on cell apoptosis activation. DEX stimulation in lv-miRC-expressing human osteoblasts resulted in robust viability (MTT OD) reduction (Fig. 4A), which was largely attenuated in the lv-pre-miR-4523-expressing human osteoblasts (Fig. 4A). Furthermore, in human osteoblasts miR-4523 overexpression significantly inhibited DEX-induced activation of caspase-3 (Fig. 4B) and caspase-9 (Fig. 4C) as well as cleavages of caspase-3 and PARP (Fig. 4D). In addition, DEX-induced accumulation of histone-bound DNA was significantly alleviated by miR-4523 overexpression (Fig. 4E).

Moreover, DEX treatment induced significant apoptosis activation in lv-miRC-expressing human osteoblasts, evidenced by increases in TUNEL-positive nuclei ratio (Fig. 4F) and Annexin V-positive cell ratio (Fig. 4G). Significantly, apoptosis activation was largely attenuated in lv-pre-miR-4523-expressing osteoblasts (Fig. 4F and G). In lv-miRC-expressing hFOB1.19 osteoblastic cells, DEX similarly induced viability (MTT OD) reduction (Fig. 4H), and apoptosis activation (TUNEL-positive nuclei ratio increase, Fig. 4I). Such actions were largely inhibited in lv-pre-miR-4523-expressing hFOB1.19 cells as well (Fig. 4H, I). These results clearly showed that miR-4523 overexpression ameliorated DEX-induced apoptosis activation in human osteoblasts.

### miR-4523-induced osteoblast cytoprotection against DEX requires activation of PGK1-Nrf2 cascade

**Fig. 5:**
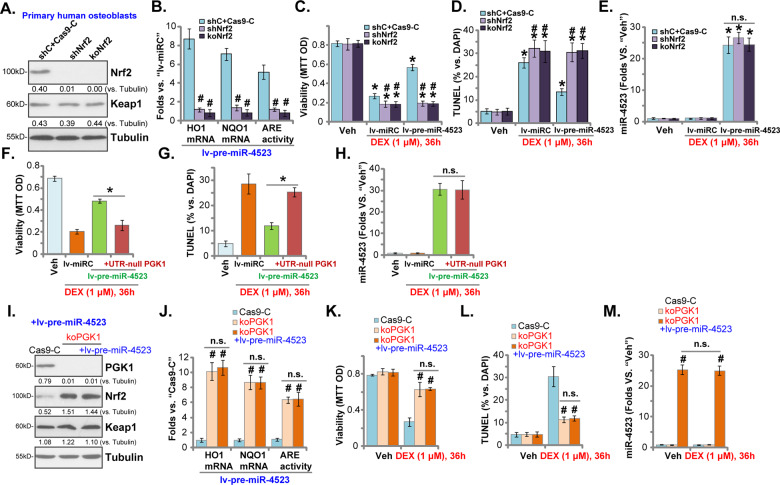
miR-4523-induced osteoblast cytoprotection against DEX requires activation of PGK1-Nrf2 cascade. Stable human osteoblasts with Nrf2 shRNA (shNrf2), a CRISPR/Cas9-Nrf2-knockout construct (“koNrf2”), or the CRISPR/Cas9 sgRNA control construct plus scramble control shRNA lentivirus (“shC+Cas9-C”) were established. These osteoblasts were further infected with pre-miR-4523-expressing lentivirus (“lv-pre-miR-4523”), with stable osteoblasts established following puromycin selection. Expression of listed genes was tested by western blotting (**A**) and qRT-PCR (**B**) assays, with relative ARE activity examined as well (**B**). Alternatively, these osteoblasts were transduced with lv-pre-miR-4523 or the control lentiviral miR construct (lv-miRC), and treated with or without DEX (1 μM) for applied time periods. Cell viability (MTT OD, **C**), apoptosis activation (TUNEL-positive nuclei ratio, **D**), and miR-4523 expression (**E**) were examined. Human osteoblasts with lv-miRC, lv-pre-miR-4523, or lv-pre-miR-4523 plus 3′-UTR-depleted PGK1 construct (“+UTR-null PGK1”) were treated with or without DEX (1 μM) for applied time periods. Cell viability (MTT OD, **F**), apoptosis activation (TUNEL-positive nuclei ratio, **G**), and miR-4523 expression (**H**) were examined. Stable human osteoblasts with the CRISPR/Cas9-PGK1-knockout construct (“koPGK1”) were further infected with or without lv-pre-miR-4523, control cells were with the CRISPR/Cas9 sgRNA control construct (“Cas9-C”). Expression of listed genes was tested by western blotting (**I**) and qRT-PCR (**J**) assays, with relative ARE activity tested as well (**J**). Cells were further treated with or without DEX (1 μM) for applied time periods; cell viability (MTT OD, **K**), apoptosis activation (TUNEL-positive nuclei ratio, **L**), and miR-4523 expression (**M**) were examined. Data were presented as mean ± standard deviation (SD, *n* = 5). *P < 0.05 versus “Veh” treatment (**C**–**E**); ^#^*P* < 0.05 versus “shC+Cas9-C” cells (**B**–**E**). *P < 0.05 (**F**–**H**). ^#^*P* < 0.05 versus “Cas9-C” cells (**J**–**M**). “n.s.” stands for non-statistical difference (**E**, **J**–**M**). The experiments were repeated five times with similar results obtained.

In order to study whether Nrf2 activation is required for miR-4523-induced cytoprotective activity in DEX-stimulated osteoblasts, Nrf2 shRNA lentiviral particles were transduced to primary human osteoblasts to establish Nrf2-silenced stable osteoblasts (“shNrf2” osteoblasts). Alternatively, a CRISPR/Cas9-Nrf2-KO-GFP construct was transfected to human osteoblasts. Through FACS sorting of GFP-positive cells and Nrf2-KO screening, single stable cells (namely“koNrf2” osteoblasts) were established. As shown, lv-pre-miR-4523-induced Nrf2 protein stabilization (Fig. 5A), ARE activity increase (Fig. 5B), as well as expression of Nrf2-dependent genes (*HO1* and *NQO1*) (Fig. 5B), were almost completely blocked in shNrf2 and koNrf2 osteoblasts. After Nrf2 silencing or KO, DEX-induced viability reduction (Fig. 5C) and apoptosis (TUNEL-positive nuclei ratio increase, Fig. 5D) were significantly intensified. More importantly, miR-4523 overexpression, by lv-pre-miR-4523, failed to inhibit DEX-induced cytotoxicity in Nrf2-depleted human osteoblasts (Fig. 5C, D). Nrf2 shRNA or KO, as expected, did not affect miR-4523 expression in lv-pre-miR-4523-expressing osteoblasts, with or without DEX treatment (Fig. 5E). These results showed that Nrf2 silencing or KO abolished miR-4523-induced osteoblast cytoprotection against DEX.

Significantly restoring PGK1 expression by the UTR-null PGK1 construct (see Fig. 2) almost abolished lv-pre-miR-4523-induced osteoblast cytoprotection against DEX (Fig. 5F, G). These results supported that PGK1 silencing should be the primary mechanism of miR-4523-induced cytoprotective activity in human osteoblasts. Again, lv-pre-miR-4523-induced miR-4523 overexpression was not affected by the UTR-null PGK1 construct (Fig. 5H).

We further hypothesized that PGK1 depletion should mimic miR-4523-induced actions. To test this hypothesis, a CRISPR/Cas9-PGK1-KO construct (see our previous study [9]) was stably transduced to human osteoblasts, resulting in complete PGK1-KO (“koPGK1” osteoblasts, Fig. 5I). The Nrf2 protein (Fig. 5I), ARE activity (Fig. 5J), as well as expression of *HO1* and *NQO1* mRNAs (Fig. 5J), were robustly increased in koPGK1 osteoblasts. In line with our previous findings [9], CRISPR/Cas9-induced PGK1-KO largely attenuated DEX-induced viability reduction (Fig. 5K) and apoptosis (Fig. 5L) in human osteoblasts. Importantly, miR-4523 overexpression by lv-pre-miR-4523 (Fig. 5M) failed to further increase Nrf2 cascade activation (Fig. 5I, J); nor did it offer further osteoblast cytoprotection against DEX (Fig. 5K and L). Unsurprisingly, PGK1-KO did not alter lv-pre-miR-4523-induced miR-4523 overexpression in human osteoblasts, with or without DEX treatment (Fig. 5M). These results implied that miR-4523 was invalid in PGK1-KO osteoblasts, further supporting that PGK1 silencing and subsequent Nrf2 signaling activation are the primary mechanisms of miR-4523-induced osteoblast cytoprotection against DEX.

### miR-4523 is downregulated in human necrotic femoral head tissues

**Fig. 6:**
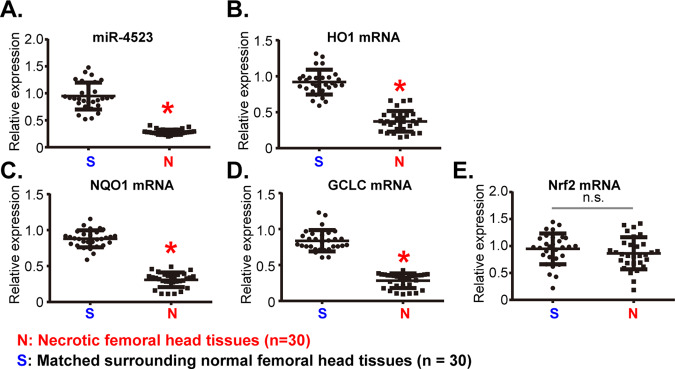
miR-4523 is downregulated in human necrotic femoral head tissues. Expression of miR-4523 (**A**) and listed mRNAs (**C**–**E**) in necrotic femoral head tissues (“N”) and surrounding normal femoral head tissues (“S”) of 30 different DEX-taking patients were examined by qRT-PCR assays. Data were expressed as mean ± standard deviation (SD, *n* = 30). *P < 0.05 vs. “S” tissues. “n.s.” stands for non-statistical difference (**E**). Experiments in this figure were repeated three times, and similar results were obtained.

At last, we examined the expression of miR-4523 in the necrotic femoral head tissues that were from DEX-taking human patients. qRT-PCR assays were employed to examine miR-4523 expression in human tissue lysates. As shown in Fig. 6A, miR-4523 levels in the necrotic femoral head tissues (“N”) were significantly lower than those in the surrounding normal bone tissues (“S”). mRNA levels of *HO1*, *NQO1,* and *GCLC*, the indicators of Nrf2 cascade activation, were downregulated in the necrotic femoral head tissues (Fig. 6B–D). Expression of *Nrf2* mRNA was however unchanged (Fig. 6E). Therefore, miR-4523 is downregulated in human necrotic femoral head tissues, correlating with Nrf2 cascade inhibition.

## Discussion

Recent studies have shown that multiple miRNAs could efficiently activate Nrf2 signaling and inhibit oxidative injury in different human cells. In human retinal pigment epithelium cells and retinal ganglion cells, microRNA-141 (miR-141) silenced Keap1 to activate the Nrf2 signaling cascade, suppressing ultraviolet (UV) radiation-induced oxidative stress and cell death [37]. Similarly, Shi et al. [38] found that Keap1 silencing by miR-141 activated Nrf2 signaling to protect hepatocellular carcinoma cells from 5-fluorouracil-induced cell death. Li et al. [39] discovered that microRNA-941 (“miR-941”) silenced Keap1 to activate the Nrf2 cascade, thereby protecting endometrial cells from oxygen and glucose deprivation-re-oxygenation-induced oxidative injury. Chen et al. [30] reported that by targeting Cul3, the ubiquitin E3 ligase of Nrf2, microRNA-601 (miR-601)-activated Nrf2 signaling to protect retinal cells from H_2_O_2_. These results implied that silencing Nrf2’s negative regulators by specific miRNAs could be a novel strategy to activate Nrf2 signaling.

In human osteoblasts, however, only a few miRNAs were reported to activate Nrf2 signaling and inhibit DEX-induced oxidative injury. Liu et al. [7] found that targeted silencing of tuberous sclerosis complex 1 by microRNA-19a (“miR-19a”) activated mTOR-dependent Nrf2 signaling cascade, inhibiting DEX-induced ROS production and oxidative injury in human osteoblasts. Zhao et al. [6] discovered that microRNA-200a (“miR-200a”) activated Nrf2 signaling by selectively silencing Keap1, protecting osteoblasts from DEX-induced oxidative injury. Zhuang et al. [24] reported that microRNA-107 inhibition increased calcium-binding protein 39 (CAB39) expression and activated AMP-activated protein kinase (AMPK)-dependent Nrf2 signaling, protecting osteoblasts from DEX.

PGK1 was reported to negatively regulate Nrf2 cascade activation [19]. Conversely, PGK1 silencing or inhibition could cause accumulation of the reactive metabolite methylglyoxal [19, 20]. The latter can form the MICA in Keap1, causing Keap1 dimerization, Keap1–Nrf2 separation, and robust Nrf2 signaling activation [19, 20]. We have previously shown that PGK1 shRNA/KO robustly activated Nrf2 signaling to ameliorate DEX-induced oxidative injury in human osteoblasts [9]. We therefore in this study explored the potential PGK1-targeting miRNAs on Nrf2 signaling cascade activation and Dex-induced osteoblast injury.

The biological functions of miR-4523 are largely unknown. We here discovered that miR-4523 is a novel and specific PGK1-targeting miRNA. RNA-FISH, RNA pull-down, and Ago2 RNA-IP experiments demonstrated that miR-4523 directly associated PGK1 in primary human osteoblasts and hFOB1.19 osteoblastic cells. Ectopic overexpression of miR-4523 decreased PGK1 3′-UTR activity, resulting in dramatic downregulation of PGK1 mRNA and protein. Importantly, PGK1 silencing by ectopic overexpression of miR-4523 activated the Nrf2 signaling cascade, causing Keap1–Nrf2 disassociation, Nrf2 protein stabilization, and its nuclear translocation as well as transcription activation and increased expression of Nrf2-dependent genes (*NQO1*, *GCLC,* and *HO1*) in human osteoblasts and hFOB1.19 cells. Conversely, miR-4523 silencing, via lv-antagomiR-4523, increased PGK1 3′-UTR activity and its expression in human osteoblasts. As a result, ARE activity was decreased in lv-antagomiR-4523-expressing human osteoblasts.

Our results implied that miR-4523-mediated PGK1 silencing activated Nrf2 signaling to protect human osteoblasts from DEX. Nrf2 silencing (by shRNA) or KO (by CRISPR/Cas9 method) completely abolished miR-4523 overexpression-induced osteoblast cytoprotection against DEX. Significantly, restoring PGK1 expression, by the UTR-null PGK1 construct, abolished Nrf2 cascade activation by miR-4523 overexpression. Furthermore, miR-4523 overexpression-induced osteoblast cytoprotection was largely attenuated with PGK1 re-expression. Conversely, CRISPR/Cas9-induced PGK1-KO resulted in constitutive Nrf2 cascade activation and protected human osteoblasts from DEX-induced oxidative injury. Intriguingly, miR-4523 overexpression failed to offer additional cytoprotective activity against DEX in PGK1-KO human osteoblasts. These results clearly showed that PGK1 silencing and subsequent Nrf2 cascade activation should be the primary mechanism of miR-4523-induced osteoblast cytoprotection against DEX.

In the present study, we found that miR-4523 levels in necrotic femoral head tissues were significantly lower than those in normal bone tissues. Further studies will be needed to explore the potential pathological mechanisms of miR-4523 downregulation by DEX. Long non-coding RNAs (LncRNAs) are transcripts exceeding 200 nucleotides [40–42] that could regulate gene expression by sponging miRNAs [43]. Our preliminary studies have identified several miR-4523-targeting LncRNAs with unknown functions. In the following studies, we will explore the expression and potential functions of these miR-4523-targeting LncRNAs in DEX-induced oxidative injury and death in osteoblasts. Whether DEX could affect the biogenesis, transcription, and maturation of miR-4523 in osteoblasts will also be tested. Our results implied that increasing miR-4523 activity could be a promising therapeutic strategy for the treatment of DEX-associated osteoporosis and osteonecrosis.

## Conclusion

PGK1 silencing by miR-4523 protected human osteoblasts from DEX through activation of the Nrf2 signaling cascade.

## Supplementary information

Figure S1

## Data Availability

The data are included in the article and its supplementary file. Further inquiries can be directed to the corresponding authors.
