# Supplementary material for: Phosphoglycerate kinase 1 silencing by a novel microRNA microRNA-4523 protects human osteoblasts from dexamethasone through activation of Nrf2 signaling cascade
Source: Cell Death Dis. 2021 Oct 19;12(11):964. doi: 10.1038/s41419-021-04250-1 (PMC8526604; doi:10.1038/s41419-021-04250-1)
Supplement: Supplementary file 1 — Figure S1 [file 41419_2021_4250_MOESM1_ESM.pdf]

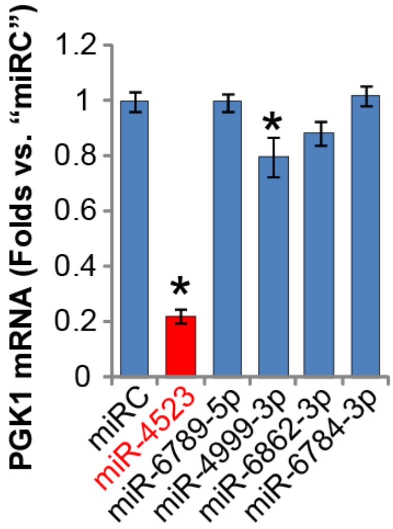

Figure **S1**. The primary human osteoblasts were transfected with applied miRNA mimics (500 nM each for 24h, two rounds) or the non-sense control miRNA mimic ("miRC", 500 nM for 24h, two rounds), expression of *PGK1* mRNA was shown. Data were presented as mean ± standard deviation (SD, n=5). * ***P*** < 0.05 versus "miRC" cells.
